# Biological performance evaluation of graphene nanoplatelets for intracranial direct current stimulation

**DOI:** 10.3389/fnins.2026.1664084

**Published:** 2026-06-25

**Authors:** Sanchit Rathi, Jakub Krzemiński, T. Vighneshvel, Andrzej Pepłowski, Dominik Baraniecki, Nikhil Tiwari, Anna Karpova, Daniel Janczak, Małgorzata Jakubowska, Michael Brosch

**Affiliations:** 1 Research Group Comparative Neuroscience, Leibniz Institute for Neurobiology, Magdeburg, Germany; 2 Centre for Advanced Materials and Technologies, Warsaw University of Technology, Warsaw, Poland; 3 Faculty of Mechanical and Industrial Engineering, Warsaw University of Technology, Warsaw, Poland; 4 Institut Für Biochemie Und Zellbiologie, Otto-Von-Guericke-Universität Magdeburg, Magdeburg, Germany; 5 Research Group Neuroplasticity, Leibniz Institute for Neurobiology, Magdeburg, Germany; 6 Center for Behavioral Brain Sciences, Otto-Von-Guericke-University Magdeburg, Magdeburg, Germany

**Keywords:** biocompatibility, cortical direct current stimulation, flexible electrodes, graphene nanoplatelets, screen-printing

## Abstract

Graphene nanoplatelets (GNPs) offer promising properties for neural interface applications, particularly in intracranial direct current (DC) stimulation, due to their high charge injection capacity, flexibility, and biocompatibility. In this study, we comprehensively evaluate the biological and functional performance of GNP-based electrodes for acute and chronic cortical stimulation and recording. GNP electrodes were fabricated via screen-printing on flexible polyimide substrates. They were assessed for cytotoxicity using RAW 264.7 macrophage cell lines and primary hippocampal cultures, showing no evidence of cell death or toxicity. Long-term implantation in non-human primates (50 days) revealed no signs of infection, inflammation, or cortical damage, supporting the biostability of the material. Magnetic resonance imaging confirmed that GNPs produce no imaging artifacts, affirming their compatibility with standard neuroimaging protocols. Functionally, epidural DC stimulation using GNP electrodes modulated neuronal firing rates in the auditory cortex in a polarity-dependent manner, consistent with classical effects of DC stimulation. Furthermore, the same electrodes reliably recorded auditory-evoked potentials, demonstrating dual functionality for stimulation and recording. These findings position screen-printed GNP electrodes as safe, versatile, and effective tools for neurophysiological applications, and highlight their potential for future use in translational neuroscience and neurotherapeutic settings.

## 1 Introduction

Electrical direct current (DC) stimulation of cortical tissue has long been employed in systems neuroscience to alter brain states. This can be achieved via two main methods: transcranial direct current stimulation, which applies weak currents through the scalp, and intracranial direct current stimulation (iDCS), which involves delivering currents directly to extracellular regions on or within the cortex. Early animal studies (Creutzfeldt et al., 1962; Bindman et al., 1964; Purpura and McMurtry, 1965) demonstrated that low-magnitude currents could modulate cortical excitability by shifting the resting membrane potential of neurons. Typically, cathodal stimulation leads to membrane hyperpolarization and a reduction in neuronal firing, while anodal stimulation causes depolarization and increased firing. The effects of DC stimulation are governed by several parameters: current magnitude, polarity, duration, and electrode size and placement (for review see Bikson et al., 2019). However, compared to alternating current stimulation, much less is known about the effects of DC stimulation on physiological states and behavior - partly due to the limited availability of suitable devices (Aplin and Fridman, 2019).

Traditionally, iDCS has employed both penetrating and non-penetrating wire electrodes (Purpura and McMurtry, 1965; Rosen and Stamm, 1972; Medeiros et al., 2012; de Souza Custódio et al., 2013). In studies involving animal subjects, glass capillary or silver-silver chloride (Ag/AgCl) electrodes have been predominantly used (Creutzfeldt et al., 1962; Rosen and Stamm, 1972). However, our preliminary tests with conventional Ag/AgCl disk electrodes for cortical DC stimulation revealed a high degree of electrolysis occuring at the electrode-tissue interface, which significantly compromised their durability and practical usability. More importantly, electrode corrosion posed a potential threat of triggering an inflammatory reaction in the cortical tissue due to the release of silver ions (Wu and Tang, 2018). This undesired electrolysis can be mitigated by employing brain stimulation electrodes fabricated using biocompatible materials with higher charge injection capacity. In recent years, studies have shown that carbon allotropes not only exhibit significantly higher charge injection capacity than conventional metallic electrodes (including platinum electrodes) but are also safer with regard to inflammatory reactions when in contact with live tissue (Vomero et al., 2016; Vomero et al., 2017). Although numerous carbon-based materials, particularly graphene, are currently being investigated or employed as coating materials in brain stimulation electrodes (Apollo et al., 2015; Park et al., 2016; Vomero et al., 2017), every electrode material must be thoroughly investigated to ensure its safety and to prevent potential tissue damage (Kumsa et al., 2017; Harris et al., 2018).

Our previous work (Pepłowski et al., 2020) demonstrated the electrochemical safety of graphene nanoplatelets (GNP)-based brain stimulation. The current study aims to evaluate the biological safety of GNP electrodes to determine their suitability for both acute and chronic brain research applications. To this end, we examined the biocompatibility of GNPs by (1) assessing cytotoxicity in cultured nerve cells and (2) monitoring brain tissue after 50 days of continuous contact with GNPs. In addition, we evaluated (3) whether GNP electrodes are compatible with non-invasive brain imaging by checking for artifacts during magnetic resonance (MR) imaging, (4) the capacity of GNP electrodes to modulate cortical activity via epidural DC stimulation, and (5) their ability to record auditory evoked potentials from the cortical surface.

## 2 Materials and methods

### 2.1 Fabrication of the graphene nanoplatelet electrode

Our previously published study (Pepłowski et al., 2020) details the fabrication procedure of GNP electrodes, including the reagents and materials used. The prototype electrodes were fabricated on a 25.4 μm thick polyimide foil Kapton^®^ 100HN (DuPont, Warsaw, Poland) serving as the base material, with a diameter of 18 mm (examples are shown in Figures 3, 5). First, holes were laser-drilled through the polyimide foil for the printing of vias between the silver conducting lines and the graphene stimulation sites. Silver paths were then screen-printed on one side of the foil. Subsequently, graphene electrodes were deposited on the other side of the foil using GNP, employing the same screen-printing method. Finally, the side with silver paths was insulated using heat-curable 8,155 paste (DuPont, Warsaw, Poland).

### 2.2 Electrochemical characterization of graphene nanoplatelet electrodes

The electrochemical properties of GNP electrodes used in this work were extensively characterized and discussed in detail in our previous publication (Pepłowski et al., 2020). The study involved cyclic voltammetry (CV), square wave voltammetry (SWV), and electrochemical impedance spectroscopy (EIS) measurements to evaluate the stability, safety and electrochemical behavior of the electrodes. Experiments were performed in phosphate-buffered saline (PBS, pH 7.2) to emulate physiological conditions. The potential window was examined between −1.0 and +1.6 V vs. Ag/AgCl to identify any undesirable redox reactions or material degradation processes.

The measurements confirmed that the graphene nanoplatelet layer itself was electrochemically stable within the −1.0 to +1.3 V range, with no significant redox activity associated with graphene or the polymer binder. The oxidation peaks observed above +1.3 V were attributed to water electrolysis, marking the upper boundary of the safe operational potential window. Further investigation identified that undesired low-potential oxidation peaks originated from partial permeability of the printed layer to the underlying silver contacts rather than from the graphene itself. Adjusting the device design to eliminate direct exposure of the silver layer effectively removed this effect. The overall results demonstrated that the printed graphene nanoplatelet electrodes are electrochemically safe for use in biological environments and suitable for direct current (DC) neural stimulation. Readers interested in detailed procedures, parameters, and discussion of electrochemical data are referred to the aforementioned study.

### 2.3 Mechanical properties of the graphene nanoplatelet electrode

The mechanical robustness of the printed GNP electrodes was evaluated by a minimum bending radius test, in order to assess the integrity and electrical stability of the conductive layer under severe deformation. Each sample was bent to a radius of 1 mm and held for 2 min, corresponding to the maximum mechanical strain expected during device handling or integration.

Electrical resistance was measured before and immediately after bending. The mechanical stability was quantified by calculating the relative resistance change, defined as ΔR/R_0_, and calculated as the percentage increase of the post-bending resistance with respect to its initial value. Additionally, to verify in any surface damage or delamination of the GNP layer occurred scanning electron microscopy (SEM) imaging was conducted with Hitachi SU-8230 SEM device. Images were acquired at an accelerating voltage of 15 kV. To increase surface conductivity and minimize charging under the electron beam, GNP electrodes were sputter-coated with a layer of gold-palladium alloy (Au:Pd 80:20) using a sputter coater (Quorum Q150T).

### 2.4 Adhesion tests of the graphene nanoplatelet electrode

Adhesion tests were carried out on square graphene nanoplatelet (GNP) test prints according to ISO 2409. Perpendicular cross-cuts were performed using a circular NT cutting knife manufactured by Testan. A grid was created on the printed area, after which the coating was cleaned with a soft brush and adhesive tape was applied in accordance with the standard.

The tape used for the tests was 1539M0025 transparent pressure-sensitive adhesive tape compliant with ISO 2409. The tape was removed after 5 min at a 60° angle. Adhesion quality was evaluated using a scale from 0 to 5, where 0 indicates no material detachment; 1, detachment of less than 5% of the total material; 2, 5%–15%; 3, 15%–35%; and 4, 35%–65% of the total pattern. Detachment greater than 65% was classified as 5.

The surface of each sample after testing was examined using a Keyence VHX-900F digital microscope and a Hitachi SU8230 scanning electron microscope (SEM). Secondary electron (SE) signals were used to determine whether the layer had been damaged and to identify any material loss.

### 2.5 Preparation of RAW 264.7 cell culture

To assess the biocompatibility of the GNP electrode, cytotoxicity tests were performed using RAW 264.7 cells. First, RAW 264.7 cells were thawed in a T-25 flask in DMEM medium supplemented with 10% FBS and glutamine. When the cell became 80% confluent, the culture was split in a T-75 flask for expansion. The GNP electrode was sterilized using an autoclave and placed inside a well of a 12-well plate. Once the cell culture in T-75 attained desired confluency, the cells were finally seeded on the GNP electrode. Two wells without GNP electrodes served as controls. Two days after cell adhesion, they were evaluated under a standard cell culture light microscope for cell expansion or cell death. Subsequently, cells were fixed using 4% PFA in PBS.

### 2.6 Rat primary hippocampal cultures, antibodies, immunocytochemistry, and confocal imaging

Primary hippocampal cultures were prepared from embryonic day 18–19 rat embryos following the protocol described by Banker and Goslin (1998). All animal procedures were conducted in accordance with ethical standards for animal research as defined by German law.

Dissociated hippocampal neurons were maintained in Neurobasal medium (NB; GIBCO/Life Technologies) supplemented with B27 (GIBCO/Life Technologies), L-glutamine (GIBCO/Life Technologies), and penicillin/streptomycin (PAA Laboratories, Pasching, Austria). Cultures were exposed to two independent treatments of GNPs for 24 h and, separately, two treatments for 48 h, and subsequently compared to untreated control conditions. To assess cell viability, cultures at DIV25 were incubated with propidium iodide (Sigma) for 10 min *in situ*, followed by fixation in 4% PFA for 5 min. Cells were then permeabilized using 0.1% Triton X-100 in PBS for 10 min, washed, and incubated for at least 45 min in blocking buffer containing 2% glycine, 0.2% gelatin, 2% bovine serum albumin (BSA), and 50 mM NH_4_Cl (pH 7.4). Primary antibodies were diluted in blocking buffer (60 μl per coverslip) and incubated overnight at 4 °C.

For immunocytochemical analysis, the following primary antibodies were used: rabbit anti-MAP2 (Sigma-Aldrich, #AB5622-I; RRID:AB_2800501) and guinea pig anti-GFAP (Synaptic Systems, # Cat. No. 173 308). Corresponding Alexa Fluor-conjugated secondary antibodies (Alexa Fluor 488, Merck; Alexa Fluor 647, Merck) were applied. Nuclear counterstaining was performed using 4’,6-diamidino-2-phenylindole (DAPI; Sigma, Cat. #D9564) at a dilution of 1:1000 in PBS for 10 min. Coverslips were mounted using Mowiol 4-88 (Calbiochem/Merck Chemicals Ltd., Cat. #475904). Confocal imaging was carried out as previously described by Karpova et al. (2013).

### 2.7 MR imaging

The electrodes were placed between two layers of chicken meat to simulate a realistic scenario for MR measurements, alongside a vitamin capsule used as a reference, in an MR-compatible paper tray. The measurements were carried out on a 3-Tesla scanner (Philips Achieva dStream, Best, Netherlands), using a 3D T1-weighed MP-RAGE sequence (TR = 12 ms, TE = 5.8 ms, TI = 900 ms, FOV = 16 × 16 × 4.8 cm^3^, matrix = 267 × 265 × 80, scan time = 10:10 min).

### 2.8 *In vivo* tests

#### 2.8.1 Subjects

Experiments were conducted on three adult male monkeys (*Macaca fascicularis*), which were also used in other studies. All experimental procedures complied with the EU Directive (2010/63/EU) on the protection of animals used for scientific purposes along with the approval from the animal care and ethics authority of the state of Saxony-Anhalt (Landesverwaltungsamt, Halle).

#### 2.8.2 Animal preparation

The animals had previously been implanted, for other purposes, with a head-holder device and a stainless-steel cylinder (22.5 mm outer diameter, 14 mm height) into the skull over the auditory cortex [Horsley-Clarke coordinates: A10 and D15; details in Brosch and Scheich (2008)]. The cylinder could be sealed with a removable and air-tight lid to protect the brain when not in use. These implants provided atraumatic head restraint and allowed for placement of the GNP electrode as well as insertion of microelectrodes into the cerebral cortex to record brain signals.

#### 2.8.3 Implantation, neuronal recordings, and electrical stimulation

Implantation of the GNP electrodes, as well as stimulations and recordings performed through them, were carried out in an electrically shielded and sound-attenuated double-walled room (1202-A, IAC). To access the brain, the animals were head-restrained, after which the lid in the implanted cylinder was opened and its interior was flushed several times with sterile 0.9% saline.

For prolonged implantion, the GNP electrode, a thin acrylic disk with a diameter slightly smaller than that of the cylinder, and a metallic C-clip were desinfected in 70% alcohol. The GNP electrode was affixed over the dura in monkey *Be*, secured by the acrylic disk, and held in place with the C-clip. Finally, the cylinder was sealed with the lid. Fifty days later, the GNP electrode was removed from the cylinder following a similar procedure.

To test the effects of current application to the brain and their usefulness for epidural recordings of the electrocorticogram from the brain within the cylinder implant, the GNP electrode was temporarily affixed over the dura with the help of a sterile acrylic disk during experimental sessions. The disk contained a small opening to grant access to the cortex. A multitrode was fitted onto a single microdrive (both from Thomas Recording, Giessen, Germany) which was then directly mounted on the cylinder using a custom-made adaptor. The multitrode was 300 μm in diameter and featured eight recording sites, each 40 μm in diameter, with an interspacing of 125 μm and impedance ranging from 0.9 to 2.3 MΩ. It was advanced into the cortex through the opening in the disk with the aid of a stainless-steel guide tube. Neuronal signals from the eight channels were pre-amplified using Thomas Recording’s pre-amplifier PA-08, band-pass filtered between 0.1 and 9 kHz, and then fed into an A/D data acquisition system (Neuralynx Inc., Bozeman, United States) at a sampling rate of 42 kHz. Multiunit activity was isolated on Cheetah32 data acquisition system based on threshold crossings and spike duration. Time-stamps of all the action potentials along with their corresponding waveforms were stored in a data-file. Neuronal recordings targeted the primary auditory cortex, whose location was previously determined in the same animal based on anatomical landmarks (the central and lateral sulci) and the known spatial tonotopic gradient from high to low frequencies along the caudal-to-rostral axis (Hackett, 2015).

A general-purpose stimulus generator was used for current- and voltage-driven electrical stimulation (STG 4002; Multi Channel Systems, Germany). Using standard measuring leads with 2.5 mm diameter plugs and crocodile clips, the active output was connected to the GNP electrode, and the ground was connected to the stainless-steel cylinder implanted in the skull (Figure 5). Two DC intensites were used, viz., −0.5 and +0.5 mA. Each DC pulse was applied for a period of 15 s followed by 15 s without stimulation. A third condition consisted of 30 s without stimulation. All conditions were presented in a randomized order, and each condition was repeated for six times.

For epidural recordings, the GNP electrode was connected to a filter amplifier (LFP-01, Thomas Recording, Giessen, Germany, passband 1–150 Hz relative to animal mass), and signals were recorded with Neuralynx at a sampling rate of 640 Hz. Auditory stimuli consisted of 100 clicks (70 dB SPL) presented at two clicks per second.

## 3 Results

### 3.1 Mechanical bending tests of the GNP electrodes

Across six samples, the normalized resistance avarage ratio was 1.03 ± 0.03 (mean ± SD, *n* = 6), corresponding to a mean relative increase of +3 ± 3%. This value is well below the 10% acceptance threshold adopted in the study, confirming that the printed conductive layer remained electrically stable and mechanically intact after bending.

**FIGURE 1:**
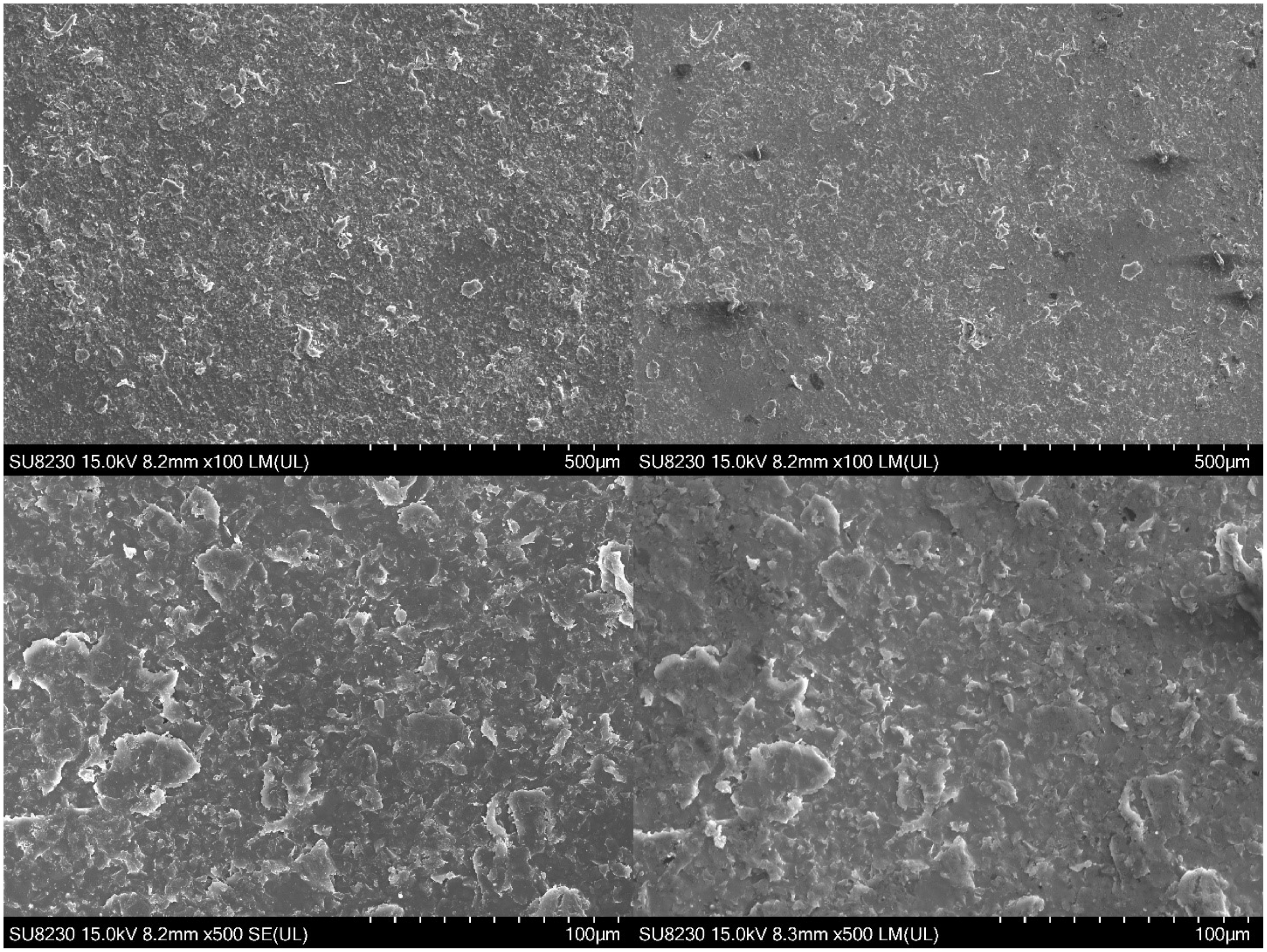
Graphene Nano-Platelet electrodes maintain structural integrity after bending. Scanning electron microscopy (SEM) images of the graphene nanoplatelet electrode before (left) and after (right) bending to a 1 mm radius for 2 min. No surface damage or delamination was observed. The mean resistance increased by only 3 ± 3% (*n* = 6), confirming high mechanical stability of the printed electrode on the flexible substrate.

Scanning electron microscopy (SEM) performed before and after bending revealed no visible cracks, delamination, or other surface degradation (Figure 1). The morphological consistency of the GNP printed layer demonstrated strong adhesion to the polymer substrate and verified the electrodes’ suitability for flexible applications on the brain.

**FIGURE 2:**
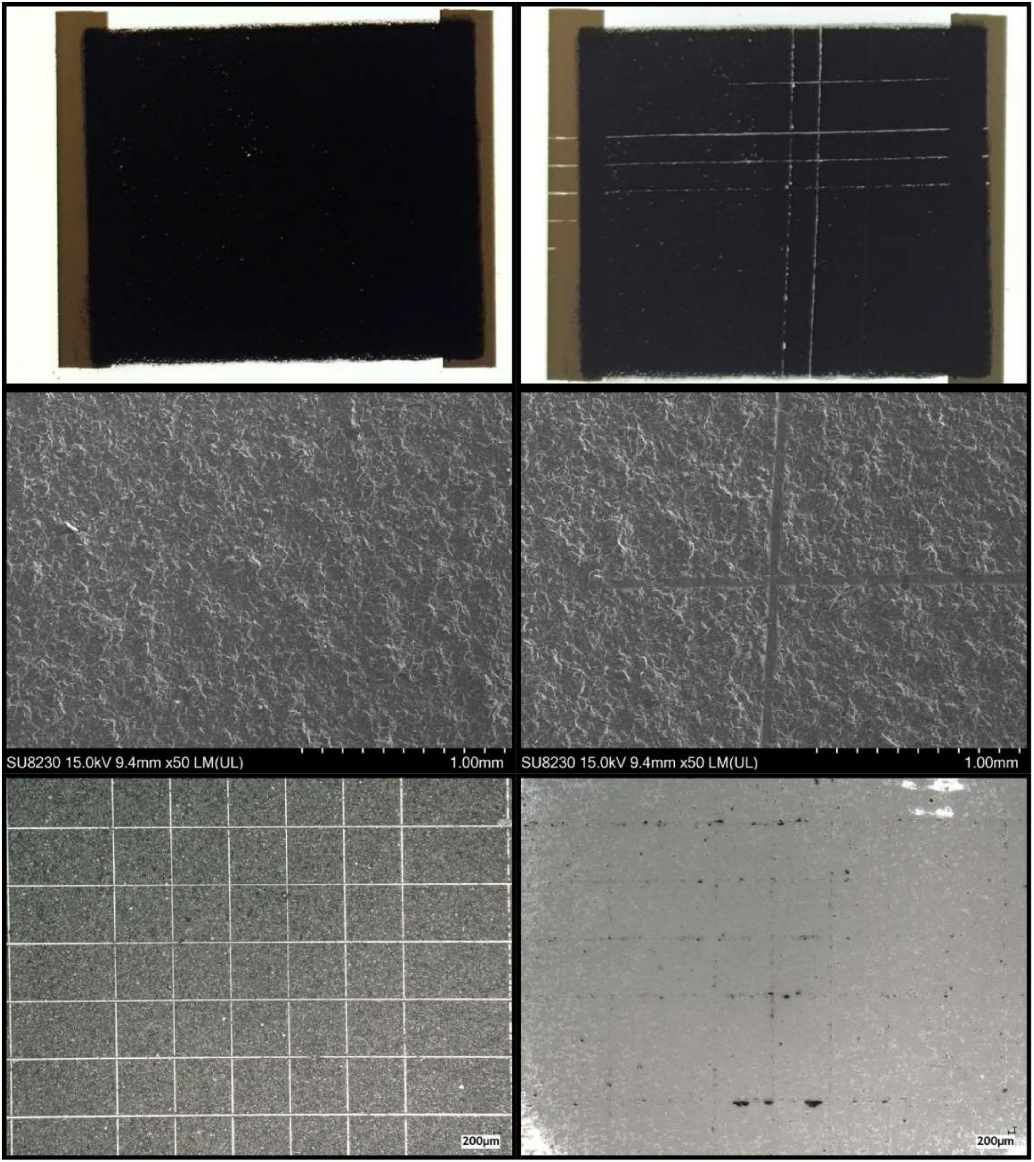
Graphene nanoplatelet electrodes adhesion test. The printed test squares before and after cutting (top row); scanning electron microscopy (SEM) images compared before (middle row, left) and after the test (middle row, right); Graphene nanoplatelet printed layer after tape delamination (bottom row, left), minimal material remains on the ISO2409 tape (bottom row, right).

Adhesion of the graphene nanoplatelet layer was evaluated on three individual samples following the ISO 2409 procedure described in the section “2 Materials and methods.” The electrodes exhibited high resistance to mechanical damage and layer detachment in the tape adhesion test (Figure 2). No significant damage to the open areas was observed in SEM images compared before and after the test.

All three samples showed consistent results, with none or only minimal detachment confined to the line-cut regions. These observations indicate that the layers were strongly adhered to the substrate, corresponding to an ISO adhesion classification of 0–1. The assigned rating may depend slightly on interpretation of the tape test, as only isolated small artifacts were present after tape removal, likely originating from the cutting process rather than adhesive failure. Therefore, the adhesion of the samples was classified as good according to ISO 2409.

### 3.2 MR imaging

**FIGURE 3:**
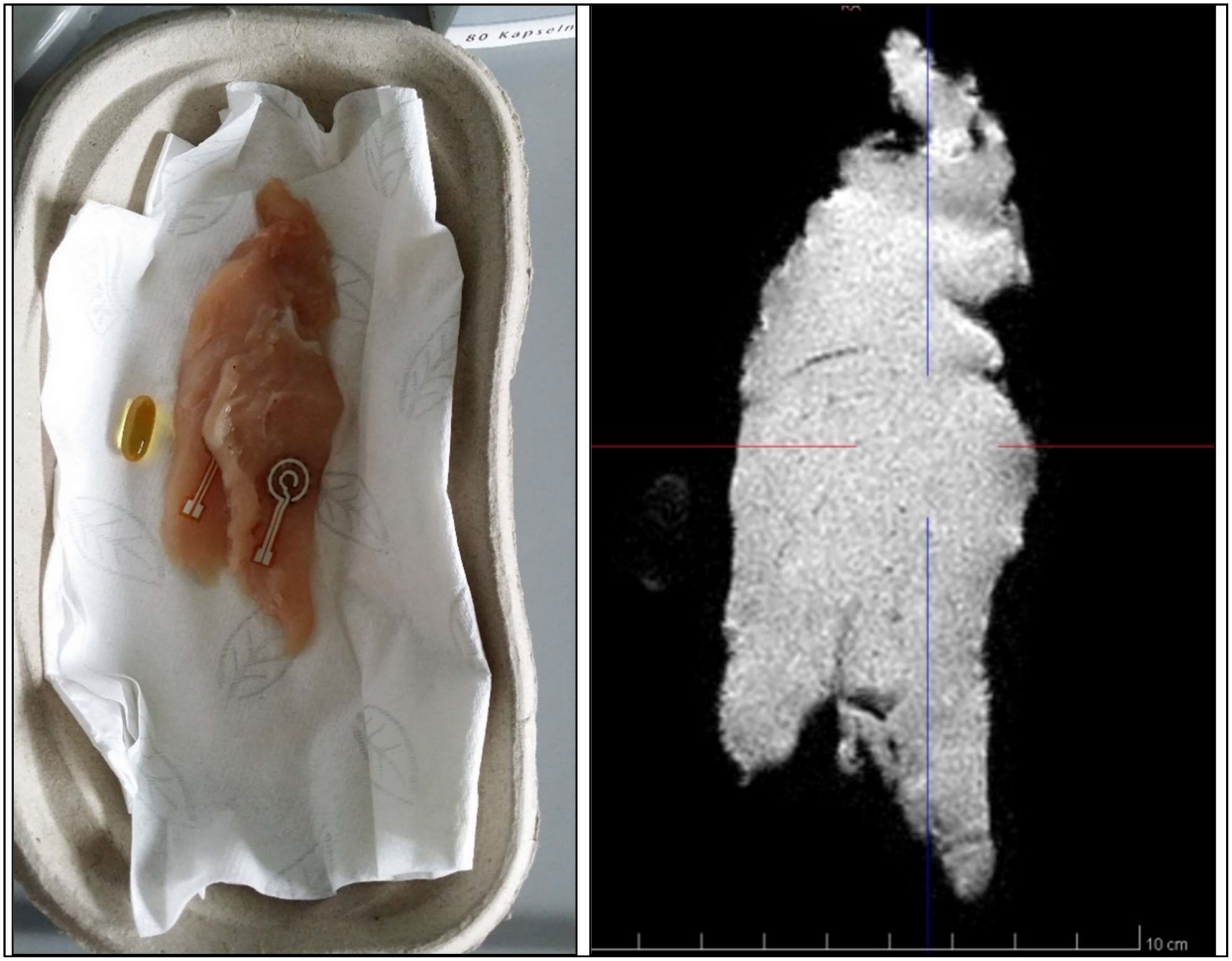
Graphene nanoplatelets do not generate artifacts during magnetic resonance (MR) Imaging. The left panel shows a photograph of an arrangement with two graphene nanoplatelet electrodes placed on top of, or between, two layers of chicken meat, alongside a vitamin capsule as a reference, all arranged in an MR-compatible paper tray. Note that in the MR image shown in the right panel, only the chicken meat is visible; the electrodes are not detectable, indicating the absence of imaging artifacts. The electrodes consisted of circular leads with diameters of 18 mm and 7 mm, a thickness of 1.5 mm, and lead wires measuring 20 mm in length.

Figure 3 shows an MR image of two GNP electrodes embedded between two layers of chicken meat. The electrodes did not generate any imaging artifacts. Moreover, no signs of heat-driven damage were observed under the MR environment. These results indicate that the GNP electrode is essentially MR-transparent as well as MR-compatible under standard imaging protocols.

### 3.3 Cell toxicity testing

**FIGURE 4:**
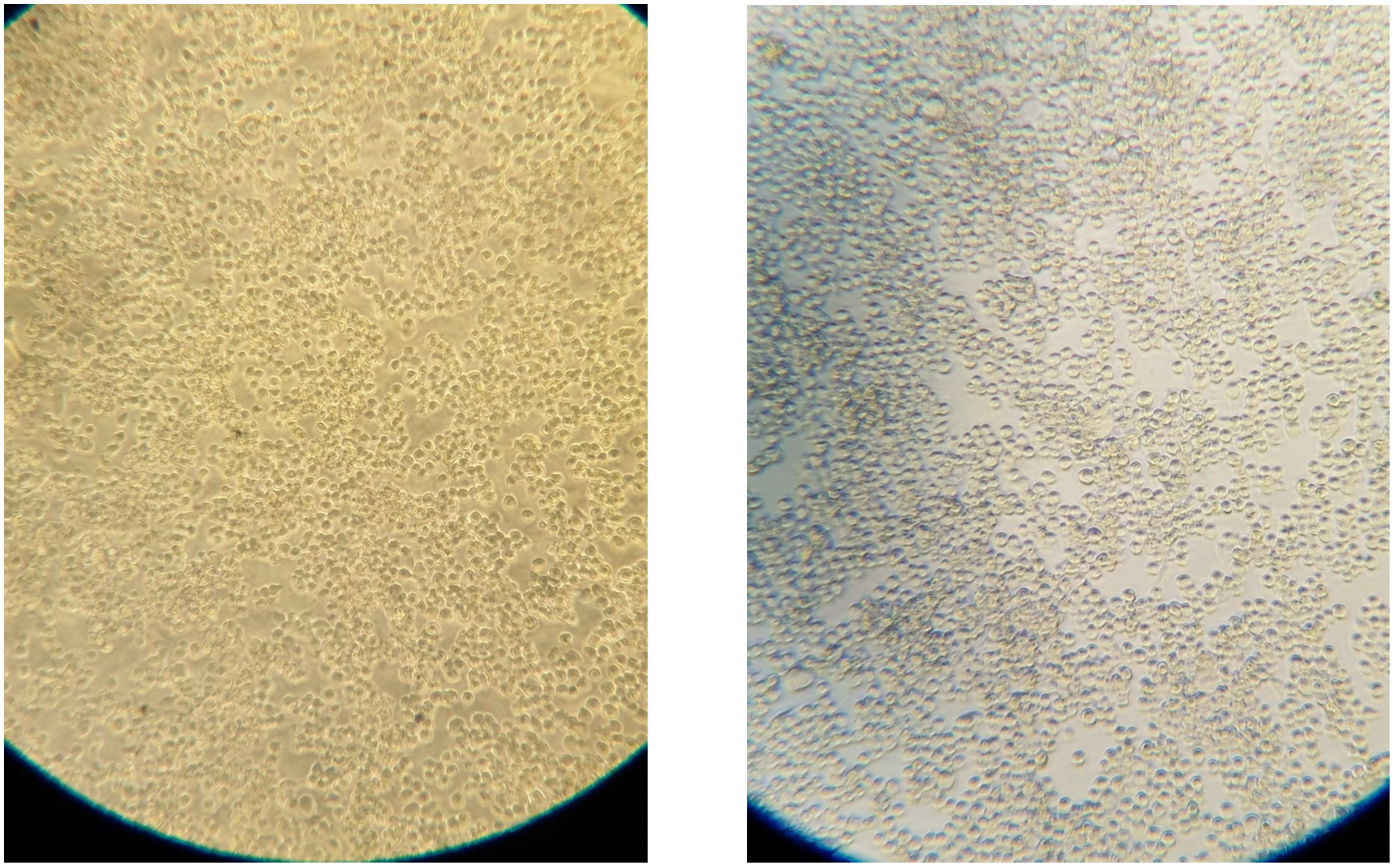
RAW 264.7 cells cultured with (left) and without (right) an graphene nanoplatelets (GNP) electrode in 12-well plates. Cell morphology and adherence appeared comparable across both conditions, suggesting good compatibility with the GNP substrate.

Figure 4 presents representative results of experiments in which equal numbers of RAW 264.7 cells were seeded in 12-well plates containing a GNP electrode and subsequently imaged using a 10 × non-immersive light microscope. Cells exhibited morphology and spatial distribution comparable to those cultured in the absence of GNP electrodes (right). The absence of a substantial population of non-adherent (floating) cells, which may include non-viable cells, was used as a qualitative indicator of apparent electrode material compatibility with biological samples. This qualitative assessment was performed across 12-wells in two independent plates.

**FIGURE 5:**
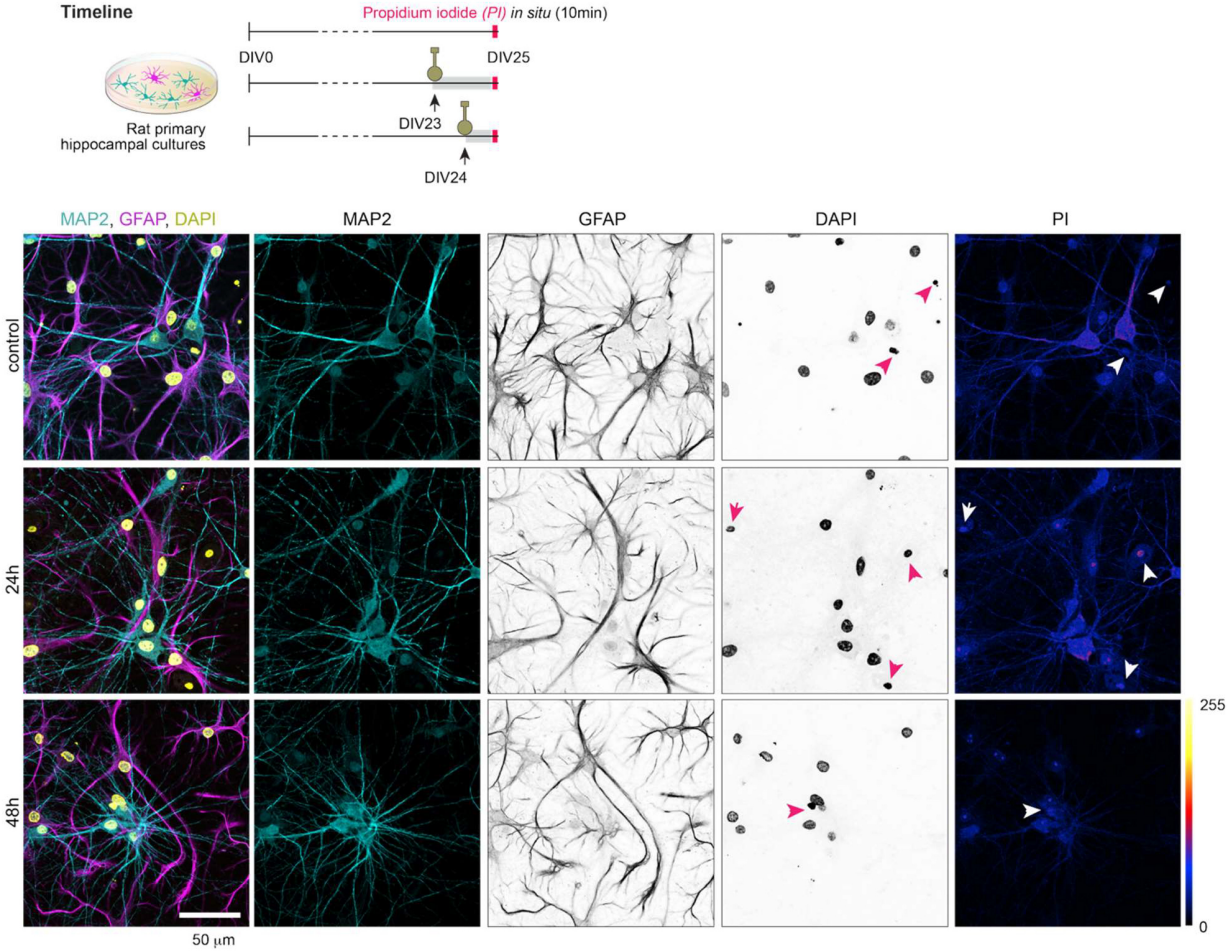
Timeline of the experiment and representative confocal immunofluorescence images of mature (DIV25) rat primary hippocampal cultures. Microtubule-associated protein 2 (MAP2), a neuronal marker, and glial fibrillary acidic protein (GFAP), an astrocytic marker, demonstrate comparable morphology and distribution of neurons and astrocytes in cultures incubated with graphene nanoplates for 24 and 48 h, compared to untreated control cultures. Propidium iodide (PI) uptake in situ, in combination with DAPI nuclear staining, revealed comparable levels of condensed PI-positive nuclei across conditions, indicating similar levels of cell death (arrows). Primary hippocampal cultures were prepared from at least 10 rat embryos. MAP2 and GFAP staining patterns remained consistent in GNP-treated cultures relative to controls.

To further assess the biocompatibility of graphene nanoplatelets in a neuronal preparation, primary rat hippocampal cultures were exposed to two independent GNP treatments for 24 h and, separately, two treatments for 48 h, as described in section “2.6 Rat primary hippocampal cultures, antibodies, immunocytochemistry, and confocal imaging.” Representative confocal images of mature cultures at DIV25 revealed comparable MAP2-positive neuronal and GFAP-positive astrocytic staining patterns in GNP-treated and untreated control cultures (Figure 5). Neuronal morphology, cellular distribution, and the overall appearance of the cultures remained unchanged after both exposure periods. In addition, propidium iodide uptake combined with DAPI nuclear staining showed similar numbers of condensed PI-positive nuclei across treated and control conditions, indicating no evident increase in cell death following GNP exposure. Together, these observations support the conclusion that exposure to GNPs does not adversely affect the survival or gross morphology of neurons and astrocytes in primary hippocampal cultures.

### 3.4 Long-term implantation effects on brain tissue

**FIGURE 6:**
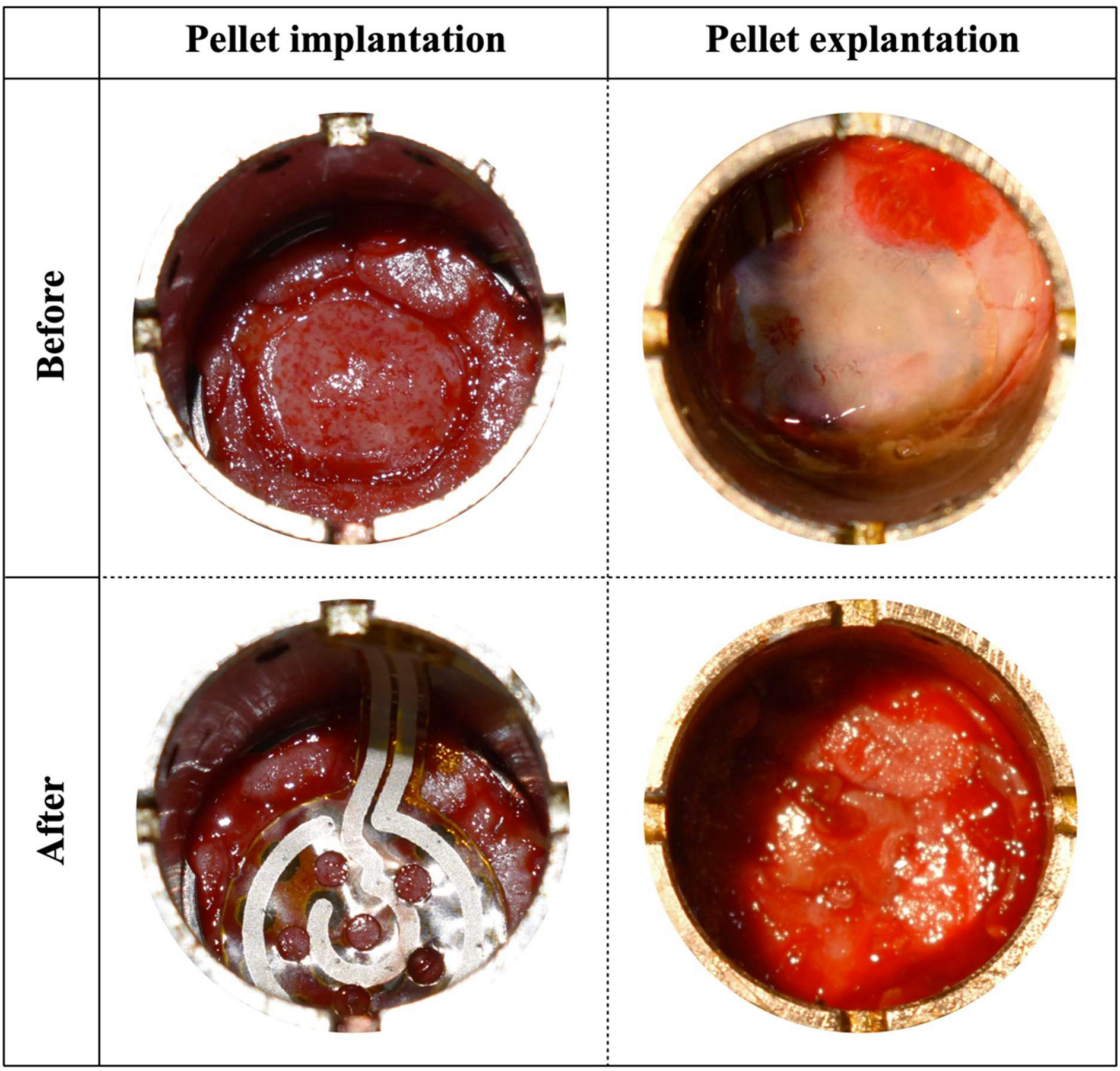
Brain surface before and 50 days after implantation of a Graphene Nano-Platelet. Each photograph shows a lateral view of the brain inside a stainless-steel cylinder. Prior to removal, only a thin layer of soft scar tissue with some vascularization was observed over the (non-visible) GNP electrode, with no signs of infection and only minor tissue changes underneath the electrode. The electrodes used had circular leads with diameters of 7 mm and 18 mm and a thickness of 1.5 mm. The cylinder had an outer diameter of 22.5 mm.

Figure 6 shows that, shortly before implantation of a GNP electrode within a stainless-steel cylinder implanted in the skull over the temporal cortex, the dura was in a healthy condition and covered by a thin layer of fibrous tissue that had gradually formed during previous intracortical microelectrode recordings. The cylinder was sealed with a lid to provide airtight protection for both the electrode and the underlying brain. Upon reopening the cylinder 50 days after implantation, a thin layer of soft tissue with some vascularization was observed covering the GNP electrode. Importantly, there were no signs of infection within the cylinder. After gentle scraping with a surgical spoon, the GNP electrode was revealed and could be easily removed from the brain surface, which was remained covered by a thin layer of fibrous tissue, similar to its condition prior to implantation.

### 3.5 Modulation of neuronal activity via epidural DC stimulation

**FIGURE 7:**
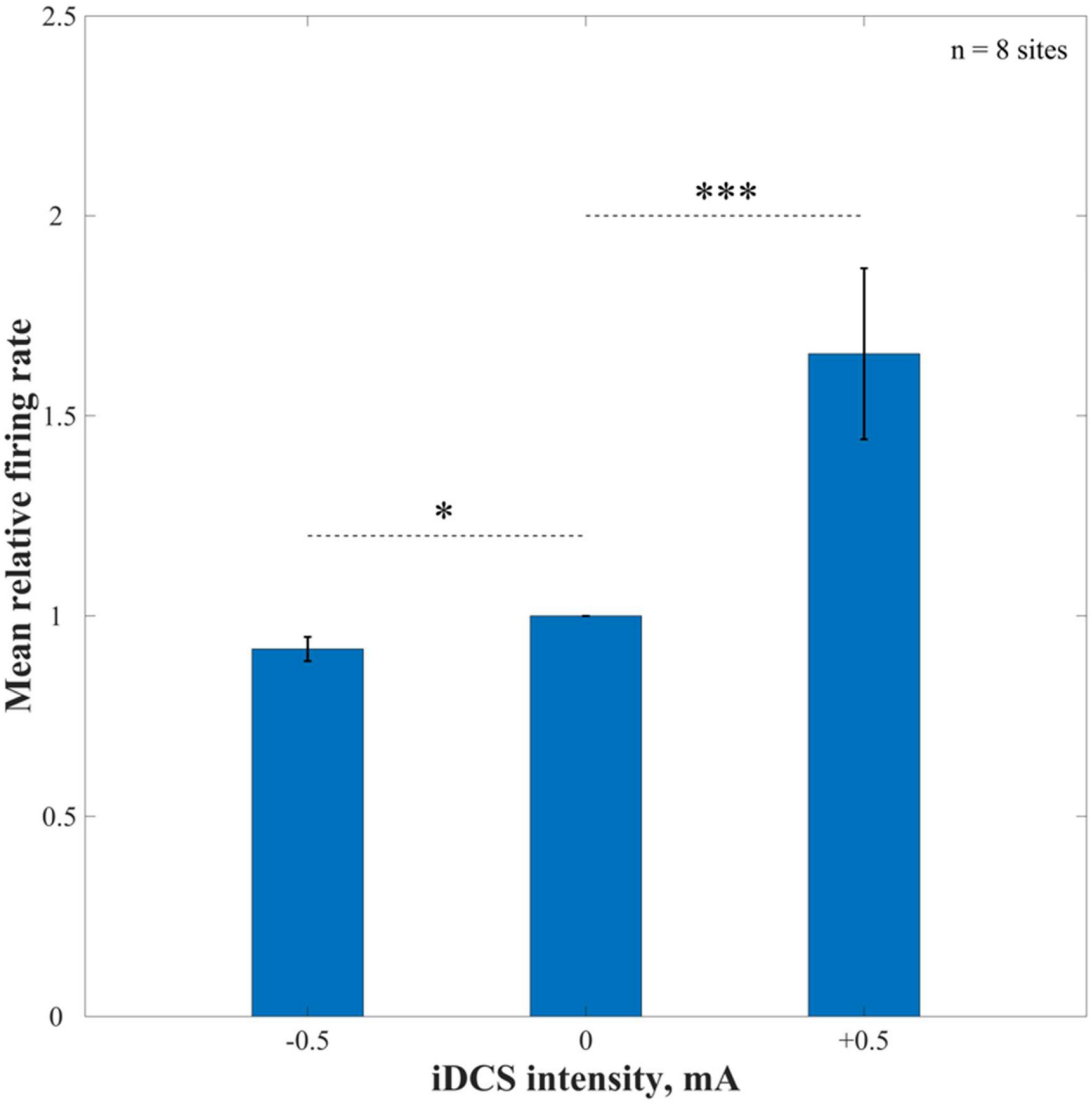
Direct currents applied through an epidural Graphene Nano-Platelet modulate resting-state neuronal firing in the auditory cortex. Mean relative firing rates of eight multiunits are plotted for two stimulation conditions and a baseline (0 mA) condition. It was defined as the ratio of firing rates during (0–15 s) to those before stimulation (–5 to 0 s). Values below 1 indicate neuronal inhibition and values above 1 indicate neuronal excitation. Asterisks denote statistical significance in the Wilcoxon signed-rank test (*p < 0.05; ***p < 0.0001) comparing each stimulation condition to the baseline.

Figure 7 shows that passing direct current through an epidural GNP electrode induces changes in the firing rates of neurons in the auditory cortex approximately 7 mm beneath the electrode. In this experiment, anodal (positive) and cathodal (negative) currents were applied for 15 s each in random order, repeated six times. Each stimulation period was followed by a 15-sinterval without stimulation. Neuronal activity from eight small clusters of neurons, i.e., multiunit activity - was recorded using an eight-channel multitrode inserted through a small cavity in the center of the GNP electrode into the brain. To quantifiy the effects of direct current for each multiunit and for each DC intensity (−0.5, 0, and +0.5 mA), firing rates were measured during the 5 s preceding stimulation and during the 15-s stimulation period. Relative firing rates were calculated as the ratio of firing rate during stimulation to the firing rate before stimulation. These relative firing rates for each DC intensity were averaged across six repetitions and subsequently across the eight multi-unit sites, and plotted against their corresponding DC intensities. Cathodal stimulation caused a significant suppression of firing rates by approximately 10% compared to the 0-mA condition (Wilcoxon signed-rank test, *p* = 0.028). Conversely, anodal stimulation significantly increased firing rates by approximately 65% (*p* = 1.5 × 10^–4^).

### 3.6 Intracranial measurement of auditory-evoked field potentials

**FIGURE 8:**
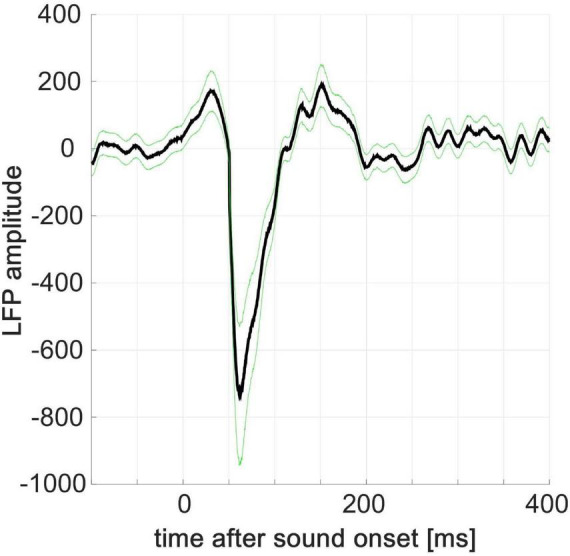
Click-evoked potential recorded using a graphene nanoplatelets (GNP) electrode placed epidurally over the auditory cortex. The thin lines indicate ± 3 standard deviations of the mean evoked potential-.

In addition to stimulation, we also evaluated the ability of the GNP electrode to record intracranial electrocorticographic signals. Figure 8 shows the average field potential recorded via an epidural GNP electrode placed above the auditory cortex within the recording cylinder during the presentation of 100 click stimuli delivered at a rate of two per second. This evoked potential consisted of a strong negative-going deflection while the presence of positive-going deflections was less clear.

## 4 Discussion

This study demonstrates that electrodes based on graphene nanoplatelets, fabricated via screen printing, are not only electrochemically effective but also biologically safe for applications in intracranial direct current stimulation and neural recording. Our results provide evidence for the biocompatibility of GNPs through in-vitro cytotoxicity assays and *in vivo* long-term implantation in non-human primates. Furthermore, GNP-based electrodes were compatible with MRI, did not generate imaging artifacts, and functioned effectively as both stimulation and recording electrodes.

The biological findings of the present study are consistent with of previous work on graphene-based materials for neural applications, where compatibility depends on material composition, surface properties, size, conductivity, and exposure duration. Earlier studies have shown that graphene can support neuronal growth and influence neuronal activity (Capasso et al., 2021), while graphene oxide has been reported to modulate astrocytic function and neuron–glia communication (Chiacchiaretta et al., 2018). Graphene-based electrodes have also demonstrated effective performance for neural recording and stimulation (Fabbri et al., 2024; Viana et al., 2024). Consistent with these reports, no obvious adverse effects of GNP exposure were observed in the present study. RAW 264.7 cultures retained normal morphology and attachment, and primary hippocampal cultures exposed to GNPs for 24 or 48 h showed staining patterns comparable to controls. These findings support the suitability of screen-printed graphene nanoplatelets for neural interfacing under the conditions tested.

A major challenge in expanding the use of iDCS in both neuroscience and clinical settings is the limited availability of electrode materials that are simultaneously flexible, biocompatible, and capable of high-charge injection (Aplin and Fridman, 2019). Traditional materials like silver/silver chloride and platinum, although commonly used, are often compromised by corrosion, electrolysis, and potential toxicity in chronic applications (Wu and Tang, 2018; Li et al., 2024). Our findings suggest that GNPs address many of these limitations, offering high-charge capacity, mechanical flexibility, and minimal inflammatory response-features that are advantageous for long-term implantation, while chronic stimulation durability under extended duty cycles requires further validation. of particularly advantageous for chronic use.

From a fabrication standpoint, screen printing enables low-cost, scalable production of flexible GNP-based electrodes. These electrodes can conform to the brain’s complex surface geometry, improving both mechanical integration and electrical performance (Apollo et al., 2015; Park et al., 2016; Vomero et al., 2016). GNP-based electrodes also exhibit superior electrochemical properties, including high-charge injection capacity (>3 mC/cm^2^) and low impedance, outperforming conventional metals like platinum (∼0.35–0.5 mC/cm^2^) and silver/silver chloride in chronic applications (Harris et al., 2018; Kumsa et al., 2017; Vomero et al., 2017; Handayani et al., 2023; Lopes et al., 2024). Functionally, the GNP electrodes successfully modulated neuronal activity during epidural DC stimulation. Cathodal currents reduced neuronal firing, while anodal currents increased it - findings consistent with the classic understanding of DC-induced polarization effects on neural excitability (Creutzfeldt et al., 1962; Purpura and McMurtry, 1965). Additionally, the ability to record auditory-evoked potentials through the same GNP electrodes highlights their dual functionality. This dual-use capability simplifies experimental setups, reduces invasiveness, and enhances the practicality of GNP-based electrodes in both research and clinical settings.

The thin, flexible nature of GNP-based electrodes also facilitates their use in long-term intracranial implantation studies, such as those investigating learning and neuroplasticity. Their MR transparency is an additional advantage, enabling simultaneous brain stimulation and imaging without introducing significant artifacts (Rodrigues et al., 2022). Moreover, the screen-printing process permits the fabrication of customized electrode shapes and arrays, supporting various experimental needs.

Comparable advances in MRI-compatible electrode design have been achieved using carbon nanotube based materials (Kanjilal et al., 2025). They share several advantageous properties with GNP electrodes compared to conventional metal electrodes, including superior electrochemical performance, mechanical flexibility, biocompatibility and high charge injection capacities, which is beneficial for both stimulation and recording. Carbon nanotube based electrodes, however, typically require specialized spinning or assembly techniques to ensure structural uniformity and reliable integration into flexible substrates. In contrast, GNP electrodes, as used in the present study, offer a more scalable and customizable alternative that simplifies large-scale, low-cost fabrication through printing processes while maintaining high conductivity and mechanical compliance.

While our study supports the safe and effective use of GNP electrodes for brain interfacing, future work should explore chronic stimulation effects beyond 50 days and investigate molecular interactions between graphene and surrounding neural and glial tissues. Longitudinal studies assessing stability, immune responses, and performance in disease models could further validate GNP-based electrodes for clinical translation.

In conclusion, our findings support the growing view that graphene-based materials represent a significant advancement in neural interface technology. GNP-based electrodes combine biocompatibility, electrochemical performance, and functional versatility, making them strong candidates for next-generation tools in both basic neuroscience and neurotherapeutic applications.

## Data Availability

The raw data supporting the conclusions of this article will be made available by the authors, without undue reservation.
